# The Impact of the COVID-19 Pandemic on Cardiovascular Diseases Hospitalizations in Brazil

**DOI:** 10.1016/j.jacadv.2024.101548

**Published:** 2025-01-16

**Authors:** Antonio Fagundes, Camila de Castro Silva, Fernanda de Sousa Rodrigues, Maria Carolina Fonseca Loureiro Caldeira de Freitas, Luiz Guilherme Passaglia, Pedro C. Pinheiro, Antonio Luiz P. Ribeiro, Bruno R. Nascimento, Debora C. Malta, Luisa C. Brant

**Affiliations:** a IDOR-D’Or Institute for Research and Education, Brasília, Distrito Federal, Brazil; b University of Brasilia-Brasília, Distrito Federal, Brazil; c Hospital das Clínicas, Universidade Federal de Minas Gerais, Belo Horizonte, Brazil; d Faculty of Medicine, Universidade Federal de Minas Gerais, Belo Horizonte, Brazil; e Department of Internal Medicine, Faculdade de Medicina, and Telehealth Center and Cardiology Service, Hospital das Clínicas, Universidade Federal de Minas Gerais, Belo Horizonte, Brazil; f Serviço de Hemodinâmica, Hospital Madre Teresa, Belo Horizonte, MG, Brazil; g Departmento Materno Infantil e Saude Publica, Escola de Enfermagem, Universidade Federal de Minas Gerais, Belo Horizonte, Brazil

**Figure:**
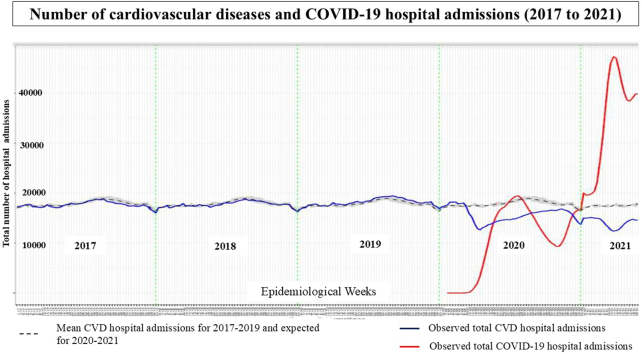

The COVID-19 pandemic was decreed by the World Health Organization on March 11, 2020, and the first confirmed case in Brazil was on February 26, 2020.^1^ Public health measures were adopted to reduce transmission and properly allocate resources.^2^ In different countries, there was a sharp decline in hospitalizations for acute cardiovascular disease (CVD) conditions during the COVID-19 pandemic.^2^ However, there is a lack of data regarding the low- and middle-income countries with large universal public health systems.

In this ecological time series study, data from public hospitals (from the SIH-Datasus)^3^ for Brazil and regions were analyzed from the epidemiological week (EW) 10 of 2020 (February 23, 2020-February 29, 2020) until the EW 21 of 2021 (May 23, 2021-May 29, 2021), defined here as the pandemic period. The study was approved by the Federal University of Minas Gerais Human Research Ethics Committee. The data used for the analyses are public and unidentified and are available.

We analyzed the impact of COVID-19 on the following outcomes: the number of hospitalizations by CVD, the number and proportion of intensive care use, and the number and proportion of in-hospital deaths. The analysis was also stratified by sex, age, and the municipality’s Human Development Index (HDI), a location’s socioeconomic development marker. HDI is composed of 3 criteria: education (illiteracy rate), longevity (life expectancy at birth), and income (gross domestic product per capita). The HDI varies from 0 to 1, 1 representing more significant socioeconomic development. For the present analysis, HDI was stratified into high (0.7-0.86), intermediate (0.62-<0.70), and low (0.42-<0.62) defined by the terciles of HDI of Brazilian municipalities.^4^

We compared the observed CVD outcomes during the pandemic to the average of the 3 previous years (2017-2019). The expected number of hospitalizations was constructed on a weekly basis based on the average of the observed number in the previous years. From the expected number by EW, we constructed the comparison period, EWs 10/2020-21/2021. The absolute differences and risk ratios (RRs) were calculated by dividing the observed values by the expected for each period studied.

**Figure 1:**
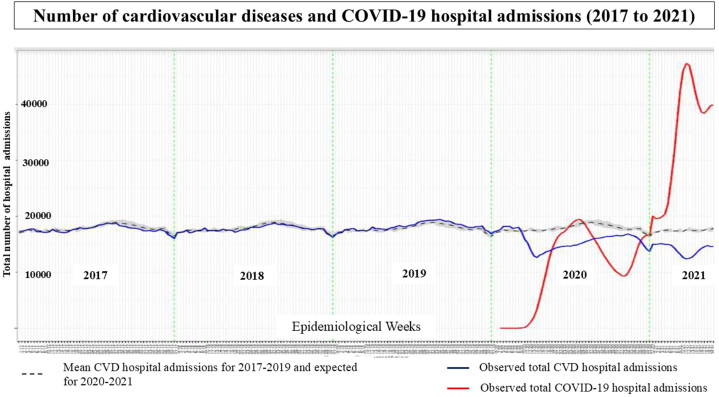
Number of Cardiovascular Diseases and COVID-19 Hospital Admissions (2017 to 2021). The Number of Cardiovascular Disease Hospital Admissions Decreased, Noticeable in 2020 (First Valley), Before the First Peak in COVID-19 Hospitalizations, and in 2021 (Second Valley), Simultaneously With the Second Peak in COVID-19 Hospitalizations The data observed were compared to the average from 2017 to 2019. The 5-week moving average of the indicators studied was compared with the corresponding average for the reference period. A particular standard errors's maximum and minimum values were used as dispersion measurements. CVD = cardiovascular disease.

There were expected 1,137,752 (95% CI: 1,125,442-1,150,063) total CVD admissions for the EWs 10 in 2020 to 21 in 2021. However, we observed 953,025 (95% CI: 928,828-977,222) CVD admissions, 184,727 (95% CI: 157,579-211,876) fewer hospitalizations than expected, representing a drop of 16.2% (RR: 0.83; 95% CI: 0.836-0.839). The first drop in CVD hospitalizations occurred before the first peak in COVID-19 hospitalizations in 2020, possibly due to adherence to social distance measures, and the second valley occurred simultaneously with the second peak in COVID-19 hospitalizations in 2021 as a reflection of competing causes (Figure 1). Furthermore, there were 216,102 (95% CI: 210,290-221,914) CVD admissions to the intensive care unit (ICU), with less 21,429 (95% CI: −27,621 to −15,237) hospitalizations than the expected 237,531 (95% CI: 235,395-239,669) revealing a 9% decrease in the number of ICU admissions (RR: 0.910; 95% CI: 0.907-0.912). Nevertheless, compared to the expected 111,495 (95% CI: 109,807-113,183) CVD admissions that resulted in in-hospital deaths, we observed a 4.7% drop, with less 5,282 (95% CI: −7,598 to −2,966) number of CVD admissions that resulted in in-hospital deaths (RR: 0.953; 95% CI: 0.949-0.957).

There was an 8.6% increase in the proportion of ICU admissions from all CVD hospitalizations (RR: 1.086; 95% CI: 1.084-1.088) and a significant 14.4% increase in the proportion of CVD hospitalizations that resulted in in-hospital deaths (RR: 1.144; 95% CI: 1.141-1.147), possibly due to greater clinical severity of hospitalized cases and disrupted care cascades. Women had a greater reduction in hospitalizations (RR: 0.813; 95% CI: 0.812-0.815) compared to men (RR: 0.858; 95% CI: 0.857-0.860), as did younger patients (<30 years: RR: 0.781; 95% CI: 0.775-0.787), compared to older age groups (30-59 years: RR: 0.849; 95% CI: 0.847-0.851; and ≥60 years: RR: 0.836; 95% CI: 0.834-0.837). Municipalities with the lowest HDI (RR: 0.819; 95% CI: 0.816-0.823) had a more significant decrease in CVD admissions than those with medium HDI (RR: 0.840; 95% CI: 0.838-0.843) or high (RR: 0.840; 95% CI: 0.839-0.841), revealing how the impact of the pandemic was affected by social determinants of health.

This study shows the overall reduction in CVD admissions during the COVID-19 pandemic in Brazil and highlights the additional negative impact on vulnerable populations such as those from municipalities with low HDI. In Brazil, public hospitals provide care to patients with low socioeconomic status regardless of the location (municipality). However, even among the low socioeconomic population, there may be a gradient in health care delivery and patients from higher HDI locations can have access to better quality care than those from lower HDI. Furthermore, we found larger reduction in CVD hospitalizations among women and young patients. For those patients admitted with CVD, a higher proportion of ICU use, and higher in-hospital mortality suggest greater disease severity in patients. Our data reinforce the CVD disparities that exist in low- and middle-income country like Brazil. In a similar analysis in India, Zachariah et al^5^ showed a 30.7% decrease in ST-segment elevation myocardial infarction admissions during COVID-19 but without an increase in in-hospital mortality, compared to prepandemic periods.^5^ Further studies are needed to identify the best strategies to create resilience in the health system so that CVD care is not disrupted, especially in vulnerable populations. Raising awareness among the population about the importance of hospital care for CVD, even during public health crises, is also needed. Understanding the importance of maintaining teams of care dedicated to CVD can minimize the impact of future pandemics.

This study has limitations. Firstly, only public health system data were evaluated. Therefore, we do not know if the behavior of the number and severity profile of admissions to private hospitals differed from those, which was beyond the scope of this analysis. In addition, despite checking the completeness of the SIH-Datasus databank, delays in filling it in are possible, although not common after this period, given that feeding the SIH database is linked to the payment of hospitalizations. Furthermore, the databank does not include baseline characteristics of the population and patient data on socioeconomic factors. In conclusion, the COVID-19 pandemic resulted in a reduction in hospitalizations for CVD in Brazil but with an increase in severity of disease, identified by a higher proportion of ICU admissions and higher in-hospital mortality. The decrease in CVD admission was not uniform. Resident in municipalities with lower HDI, a location’s socioeconomic development marker, women, and young patients had the largest reduction in CVD admissions during the COVID-19 pandemic.
